# Acclimation of Bryophytes to Sun Conditions, in Comparison to Shade Conditions, Is Influenced by Both Photosynthetic and Ultraviolet Radiations

**DOI:** 10.3389/fpls.2019.00998

**Published:** 2019-08-02

**Authors:** Gonzalo Soriano, María-Ángeles Del-Castillo-Alonso, Laura Monforte, Encarnación Núñez-Olivera, Javier Martínez-Abaigar

**Affiliations:** Faculty of Science and Technology, University of La Rioja, Logroño, Spain

**Keywords:** acclimation, bryophytes, chlorophyll, flavonoids, *Marchantia*, sclerophylly, ultraviolet radiation, xanthophylls

## Abstract

We studied the acclimation modalities of bryophytes to sun and shade under ambient or close-to-ambient conditions, measuring variables usually influenced by photosynthetically active (PAR) and ultraviolet (UV) radiations. Our aim was to elucidate to what extent the responses to changing radiations were influenced by PAR and UV wavelengths. For this aim, we used three taxonomically and structurally different species: the thalloid liverwort *Marchantia polymorpha* subsp. *polymorpha*, the leafy liverwort *Jungermannia exsertifolia* subsp. *cordifolia*, and the moss *Fontinalis antipyretica*. In the field, liverworts were more radiation-responsive than the moss, and the thalloid liverwort was more responsive than the leafy liverwort. Sun plants of *M. polymorpha* showed, in comparison to shade plants, higher sclerophylly, lower Chl *a* + *b* contents, higher Chl *a*/*b* ratios, higher (antheraxanthin + zeaxanthin)/(violaxanthin + antheraxanthin + zeaxanthin) ratios (xanthophyll index), lower *F*_v_/*F*_m_ values, higher contents of methanol-soluble vacuolar UV-absorbing compounds (soluble UVACs), higher values of the ratio between the contents of methanol-insoluble cell wall-bound UVACs (insoluble UVACs) and soluble UVACs, higher contents of soluble luteolin and apigenin derivatives and riccionidin A, and higher contents of insoluble *p*-coumaric and ferulic acids. Overall, these responses reduced light absorption, alleviated overexcitation, increased photoprotection through non-photochemical energy dissipation, increased UV protection through UV screening and antioxidant capacity, and denoted photoinhibition. *J. exsertifolia* showed moderate differences between sun and shade plants, while responses of *F. antipyretica* were rather diffuse. The increase in the xanthophyll index was the most consistent response to sun conditions, occurring in the three species studied. The responses of soluble UVACs were generally clearer than those of insoluble UVACs, probably because insoluble UVACs are relatively immobilized in the cell wall. These modalities of radiation acclimation were reliably summarized by principal components analysis. Using the most radiation-responsive species in the field (*M. polymorpha*), we found, under close-to-ambient greenhouse conditions, that sclerophylly and Chl *a* + *b* content were only influenced by PAR, *F*_v_/*F*_m_, and luteolin and apigenin derivatives were only determined by UV, and xanthophyll index was influenced by both radiation types. Thus, responses of bryophytes to radiation can be better interpreted considering the influence of both PAR and UV radiation.

## Introduction

Plants and algae are capable of efficiently absorbing and utilizing photosynthetically active radiation (PAR, 400–700 nm) to perform photosynthesis. However, excess PAR can be harmful due to photoinhibition and photo-oxidative damage to the photosynthetic apparatus. Thus, in order to absorb enough PAR for photosynthesis but, at the same time, prevent the detrimental effects of excess PAR, photosynthetic organisms acclimate to a wide range of PAR conditions. Due to spatial and temporal variations, PAR availability is continuously changing in natural environments, from full sunlight to strong shade conditions. Thus, light acclimation is a key aspect in plant physiology and many studies have been conducted on this topic for a long time. Most studies have explored plant acclimation to different PAR levels by measuring a number of response variables related to the photosynthetic apparatus and/or the photosynthesis process itself: photosynthetic pigment composition, chlorophyll fluorescence variables, photosynthesis rates, etc. (Pearcy, 1998; Esteban et al., 2015; Poorter et al., 2019).

Apart from PAR, solar radiation reaching the Earth’s surface also comprises other minor wavelengths, such as ultraviolet (UV) radiation, which represents around 6% of the total amount of solar radiation (Robson et al., 2019). UV radiation reaching the ground is distributed in two wavelength ranges: the major UV-A (315–400 nm) and the minor UV-B (280–315 nm) fractions. As PAR, UV radiation induces a number of responses which nowadays are considered rather a regulation of plant metabolism and morphology than a simple accumulation of damage (Jansen and Bornman, 2012). Thus, plants also can acclimate to UV radiation (Jansen et al., 1998), at least partially mediated by specific UV-B photoreceptors (Jenkins, 2017). UV acclimation is associated with diverse physiological responses, some of which may cause damage, such as chlorophyll degradation, photoinhibition, and decreases in photosynthetic rates (Jansen et al., 1998). However, the most frequent acclimation response of plants to increased UV radiation is the accumulation of UV-absorbing compounds (UVACs) (Searles et al., 2001; Newsham and Robinson, 2009). In tracheophytes, the chemical nature of UVACs is mainly phenolic (hydroxycinnamic acid derivatives, flavonoids, anthocyanidins, etc.), and these compounds play key roles as antioxidants and UV screens. Their location in different cell compartments (cell walls, cytosol, vacuole, endoplasmic reticulum, chloroplast, nucleus, and small vesicles) and leaf locations (such as cuticles and epidermis) may be important to properly interpret their diverse functions (Agati et al., 2012; Mouradov and Spangenberg, 2014). For example, cell wall-bound UVACs may serve as UV screens, whereas soluble vacuolar flavonoids may act as antioxidants. In bryophytes, compartmentation may be equally important from the ecological and evolutionary perspectives (Clarke and Robinson, 2008; Robinson and Waterman, 2014; Monforte et al., 2018).

Under field conditions, plant physiological responses are obviously influenced by interacting PAR, UV-A, and UV-B radiation (Barnes et al., 2005). This has also been demonstrated under controlled conditions (Götz et al., 2010). For example, exposure to UV-B under low PAR levels accentuates UV damage (Barnes et al., 2005), and a combination of PAR and UV wavelengths leads to enhanced UVACs accumulation as occurs with UV alone (Krause et al., 2003; Guidi et al., 2011). Concurrently, UV radiation can affect diverse photosynthetic variables also affected by PAR, such as chlorophyll or carotenoids contents, quantum yield, photosynthesis rates, etc. (Jansen et al., 1998; Hakala-Yatkin et al., 2010). The specific role of each wavelength on all these responses is not perfectly known. Thus, to fully understand plant acclimation to sun conditions, it is important to take into account response variables related to both the photosynthetic apparatus and processes more specifically induced by UV radiation, such as UVACs accumulation. This “global” acclimation to enhanced radiation can be important because the plasticity of certain plant traits, such as leaf pigmentation, defensive secondary metabolites, and sclerophylly (leaf mass per unit area), all of which are influenced by both PAR and UV radiation, can be crucial for predicting and managing the effects of climate change on plants.

Bryophytes are structurally simple plants with a life cycle dominated by a green gametophyte, and comprise three main evolutionary lineages: liverworts, mosses, and hornworts. Moss gametophores are always composed of a stem bearing leaves, whereas those of hornworts are always thalloid and those of liverworts can be either thalloid or leafy. Bryophytes are expanding as important model systems in plant research, not only due the use of the classic model moss *Physcomitrella patens* but also because of the emerging new model species *M. polymorpha* (liverworts) and *Anthoceros agrestis* (hornworts) (Bowman et al., 2016). In addition, bryophytes have an outstanding evolutionary relevance because they are considered to be the first true plants to have colonized land, which makes the knowledge of how they managed to cope with higher levels of PAR and UV radiation than those present in the primeval aquatic environment important. This could be inferred by studying the acclimation of extant bryophytes to sun and shade conditions in the field (Robson et al., 2019).

Bryophytes are generally seen as shade plants, but many species are well adapted to high levels of sunlight (Marschall and Proctor, 2004). Most studies on the ability of bryophytes to acclimate to sun and shade conditions have considered response variables related only to the photosynthetic apparatus (Martin and Churchill, 1982; Glime, 1984; Rincón, 1993; Marschall and Proctor, 2004; Hajek et al., 2009; Schroeter et al., 2012; Proctor and Smirnoff, 2015). Only a few studies have included variables more specifically related to UV radiation, such as the presence or induction of protective UVACs, but without a specific identification (Post and Vesk, 1992; Robinson et al., 2005; Hooijmaijers and Gould, 2007).

To sum up, many studies have dealt with the acclimation to sun and shade conditions in bryophytes and (particularly) tracheophytes, taking mostly (or solely) into account PAR levels and variables related to the photosynthetic apparatus. Thus, the role of UV radiation and associated response variables in the acclimation to sun conditions, in comparison with shade conditions, has been underexplored. In addition, many studies on bryophytes have been performed under laboratory conditions. Thus, in this context, the aim of the present study was to identify common or species-specific response strategies of bryophytes to sun and shade under field conditions, evaluating to what extent the responses to high PAR and high UV radiation were important for their acclimation. As experimental species, we used three bryophytes of different structure and taxonomical position: the thalloid liverwort *M polymorpha* subsp. *polymorpha* (a close relative of the model liverwort *M. polymorpha* subsp. *ruderalis*), the leafy liverwort *J. exsertifolia* subsp. *cordifolia*, and the moss *F. antipyretica*. The samples studied grew completely wet, thus avoiding the interference of desiccation on their responses. We measured both basic physiological variables related to the photosynthetic apparatus and potentially UV-responding variables, such as UV-absorbing compounds. A morphostructural variable [the sclerophylly index (SI)] was also measured. Once the responses of the different species had been identified, the most radiation-responsive species in the field was used to perform an experiment under close-to-ambient greenhouse conditions to elucidate the influence of photosynthetic and UV wavelengths on its radiation acclimation.

## Materials and Methods

### Plant Material and Radiation Environment of Sampling Sites

**Figure 1:**
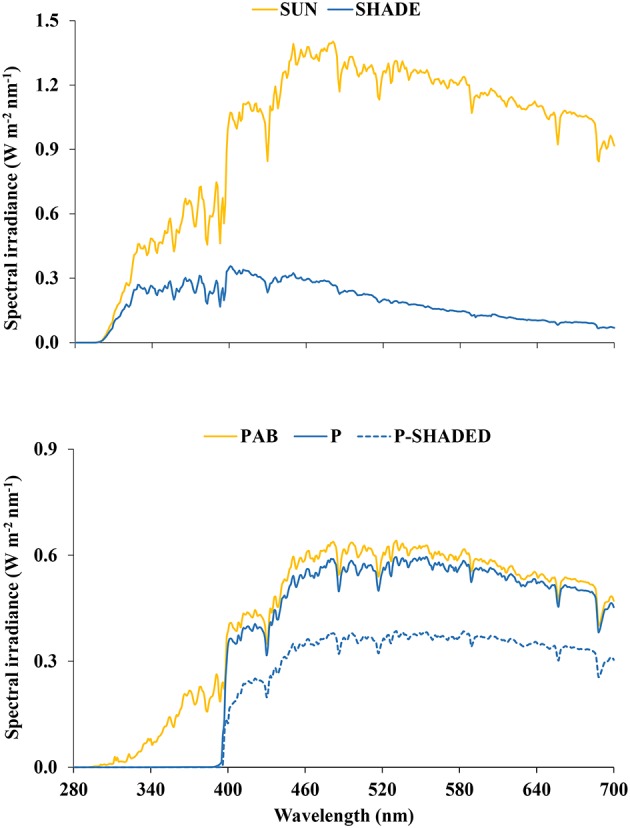
(Top) Representative spectral irradiance (280–700 nm) measured around midday on sunny days under sun and shade conditions in a typical sampling site. (Bottom) Spectral irradiance (280–700 nm) received by the thalloid liverwort *Marchantia polymorpha* subsp. *polymorpha* under the three radiation regimes imposed in the greenhouse experiment: P (PAR alone), P-shaded, and PAB (PAR + UV-A + UV-B).

Samples of three hydrophylous or hygrophilous bryophytes of different taxonomic position and structure [the thalloid liverwort *M. polymorpha* L. subsp. *polymorpha*, the leafy liverwort *J. exsertifolia* Steph. subsp. *cordifolia* (Dum.) Vaňa, and the moss *F. antipyretica* Hedw.] were collected in the basin of the river Iregua (La Rioja, northern Spain). Permanently wet or submerged (0–3 cm water depth) samples were selected to ensure a free-from-drought healthy physiological state and to homogenize the environmental conditions other than the radiation they were exposed to. Samples were collected between 950 and 1,650 m altitude, 42.04 and 42.17°N latitude, and −2.54 and −2.73°E longitude, at the central hours of the day (approximately between 12 and 15 h, solar time) on two consecutive sunny summer days (July 15–16, 2014). Ten samples of each species were collected; five of them were exposed to full sun and the remaining five were shaded by vegetation or topography. The radiation environment of the sampling sites was characterized by measuring spectral irradiance between 280 and 700 nm with a spectroradiometer (Macam SR9910, Macam Photometrics Ltd., Livingston, Scotland), placing the sensor close to the bryophyte masses. Spectra were taken around midday on comparable sunny days prior to or following the collection days. Given that the radiation spectra measured in the different sampling sites were very similar, only representative spectra of both sun and shade conditions are shown (Figure 1). The photosynthetic photon flux density (PFD) received by the samples at the time of sampling was measured using a quantum sensor (LI-190SA, LI-COR, Lincoln, NE, USA). Sun-exposed samples received around 1,400–1,500 μmol m^−2^ s^−1^, whereas shaded samples received around 200–300 μmol m^−2^ s^−1^.

### Greenhouse Experiment

Gemmae of *M. polymorpha*, which had been obtained from field-grown thalli, were cultivated in irrigated Jiffy pellets for 3 weeks in a greenhouse (23 ± 2°C day temperature, 20 ± 1°C night temperature). Radiation sources were daylight in combination with sodium lamps (Philips Master Agro 400W E40 1SL/12, Philips Lighting España, Madrid, Spain) and a Hönle SOL 1200RF2 lamp (Dr. Hönle AG UV-Technologie, Gräfelfing, Germany). The Hönle lamp was switched on around noon for 5 h per day (square-wave). Three replicates of the following three radiation regimes were set by covering the samples with specific cutoff filters:

1. P (PAR alone), using XT Vitroflex 395 Solarium Incoloro (Polimer Tecnic, Girona, Spain), which cut off UV radiation.
2. P-shaded, using XT Vitroflex 395 and a 40% white shade cloth (Macoglass, Valladolid, Spain).
3. PAB (PAR + UV-A + UV-B), using PMMA XT Vitroflex 295 (Polimer Tecnic), which cut off UV-C radiation.

The filters were pre-irradiated for 1 h and replaced after every 5 days of irradiation. The spectral irradiances were measured, and the transmission characteristics of the filters were regularly checked, with a spectroradiometer (Figure 1).

### Physiological Analyses

In the field, three replicates (100 mg each) of green and healthy apices of each species and site were collected and stored in Eppendorf tubes, which were immediately placed in liquid nitrogen to avoid metabolic changes until analysis. Chlorophyll fluorescence measurements were performed *in situ*, and the remaining variables were measured in the laboratory. The measurements taken on greenhouse-grown samples were performed in the laboratory at the end of the culture, using three replicates.

Maximum (*F*_m_) and minimum (*F*_0_) chlorophyll fluorescence values were measured after 20 min dark adaptation, with a portable pulse amplitude modulation fluorometer (MINI-PAM, Walz, Effeltrich, Germany), following Fabón et al. (2010). Then, the maximum quantum yield of PSII (*F*_v_/*F*_m_) was determined, where *F*_v_ = *F*_m_ − *F*_0_.

Photosynthetic pigments were extracted from frozen apices using 100% acetone, after grinding them in a TissueLyser (Qiagen, Hilden, Germany). Pigments were analyzed using an Agilent HP1100 HPLC system (Agilent Technologies, Palo Alto, CA, USA) and an Agilent photodiode array detector, following Otero et al. (2006). Chlorophylls *a* and *b*, β-carotene and xanthophylls (lutein, neoxanthin, violaxanthin, antheraxanthin, and zeaxanthin) were determined and quantified using commercial standards of chlorophylls *a* and *b* (Fluka), and lutein, zeaxanthin and β-carotene (CaroteNature). The Chl *a*/*b* ratio and the xanthophyll index (antheraxanthin + zeaxanthin)/(violaxanthin + antheraxanthin + zeaxanthin) were also calculated.

Total contents of UV-absorbing compounds (UVACs) were spectrophotometrically analyzed following Fabón et al. (2010). In brief, frozen apices were ground in a TissueLyser, and then 2 ml of methanol:water:7 M HCl (70:29:1, v/v/v) was added for extraction (24 h at 4°C in the dark). The extract was centrifuged to differentiate two UVACs fractions: the methanol-soluble UVACs (SUVACs) in the supernatant and the methanol-insoluble UVACs (IUVACs) in the pellet. Subsequently, the pellet was subjected to alkaline digestion to extract the insoluble compounds. Presumably, SUVACs are mainly located in the vacuoles whereas IUVACs are bound to the cell walls (Clarke and Robinson, 2008). Then, we measured the total contents of SUVACs and IUVACs as the area under the absorbance curve of each fraction in the interval 280–400 nm (AUC_280–400_), using an Agilent 8453 UV-Visible spectrophotometer. The ratio between the total contents of SUVACs and IUVACs (SUVAC/IUVAC) was also obtained.

Individual UVACs were analyzed by ultra-performance liquid chromatography (UPLC) using a Waters Acquity UPLC system (Waters Corporation, Milford, MA, USA), following Soriano et al. (2019b). The UPLC system was coupled to a micrOTOF II high-resolution mass spectrometer (Bruker Daltonics, Bremen, Germany) equipped with an Apollo II ESI/APCI multimode source and controlled by the Bruker Daltonics Data Analysis software. To quantify the different compounds, the following standards were used: apigenin, luteolin, coumarin, caffeic acid, *p*-coumaric acid, and ferulic acid (Sigma-Aldrich, St. Louis, MO, USA). Given that there is no commercial standard available for *M. polymorpha* riccionidins, they were identified according to their maximum absorbances at 485 nm, and UV absorbance was used for quantification.

The SI was calculated as the quotient between the dry mass (DM: 60°C for 24 h) and the surface area of the prostrate bryophyte apices onto the horizontal plane (LI-3000 area meter, LI-COR, Lincoln, NE, USA).

### Statistical Analysis

For the field experiment, the global effects of the species and exposure (sun or shade) on the responses of each physiological variable were tested using a two-way analysis of variance (ANOVA), once proved that the data met the assumptions of normality (Shapiro-Wilks’s test) and homoscedasticity (Levene’s test). In the case of significant differences, means were then compared by Tukey’s test. For each species and physiological variable, differences between sun and shade samples were tested using Student’s *t* test. In every case, three replicates of each variable were used. The samples were ordinated by principal components analysis (PCA), taking into account the variables that were common to the three species studied. For the greenhouse experiment, Student’s *t* tests were applied for each variable to test the differences between P and P-shaded regimes, and between P and PAB regimes. All the statistical procedures were performed with SPSS 24.0 for Windows (SPSS Inc., Chicago, IL, USA).

## Results

### Individual Ultraviolet-Absorbing Compounds Found in the Different Species

A total of 13 individual UVACs were identified (for convenience, compounds will be referred to as C1–C13): luteolin 7,4′-di-*O*-glucuronide (C1), apigenin 7,4′-di-*O*-glucuronide (C2), luteolin 7-*O*-glucuronide (C3), apigenin 7-*O*-glucuronide (C4), riccionidin A (C5), riccionidin B (C6), caffeoylmalic (phaselic) acid (C7), 5″-(7″,8″-dihydroxy-7-*O*-β-glucosyl-coumaroyl)-2-caffeoylmaic acid (C8), 5″-(7″,8″-dihydroxycoumaroyl)-2-caffeoylmaic acid (C9), *p*-coumaroylmalic acid (C10), feruloylmalic acid (C11), *p*-coumaric acid (C12), and ferulic acid (C13). C1–C11 and C12–C13 were identified in the soluble and insoluble fractions, respectively. C1–C6 and C12–C13 were found in *M. polymorpha*, C7–C11 and C12–C13 in *J. exsertifolia*, and C12 in *F. antipyretica*. The major soluble compounds in *M. polymorpha* and *J. exsertifolia* were C2 and C7, respectively. Riccionidins were not detected in the samples of *M. polymorpha* grown in the greenhouse.

### Influence of Species and Sun Exposure on the Physiological Responses of Field Samples

Similar variations in pigments and UVACs were found when they were expressed per DM, per surface area and (when possible) per Chl *a* content. Thus, only results on a DM basis will be described.

The global effect of both species and exposure (sun/shade) on the physiological variables of field samples was highly significant (*p* < 0.001). The species effect was stronger, being significant for most of the variables that were common to the three species studied (Table 1): the total contents of SUVACs and IUVACs, the contents of the individual UVACs that were common to the three species studied, SI (*p* < 0.001 for all these variables), and Chl *a* + *b* content and Chl *a*/*b* ratio (*p* < 0.01). The species effect was not significant for the xanthophyll index, the contents of β-carotene, lutein and neoxanthin, and *F*_v_/*F*_m_. The effect of exposure was only highly significant for the xanthophyll index, *F*_v_/*F*_m_ (*p* < 0.001), the total content of SUVACs, the SUVAC/IUVAC ratio, Chl *a* + *b* content (*p* < 0.01), and *p*-coumaric acid content (*p* < 0.05).

**Table 1:** Global effects of species and sun exposure (full sun vs. shade) on the physiological variables measured in field samples of the three species studied (*Marchantia polymorpha* subsp. *polymorpha*, *Jungermannia exsertifolia* subsp. *cordifolia*, and *Fontinalis antipyretica*).

	Species effect	Exposure effect
Chl *a* + *b*	^**^	^**^
Chl *a*/*b* ratio	^**^	ns
β-carotene	ns	ns
Lutein	ns	ns
Neoxanthin	ns	ns
Xanthophyll index	ns	^***^
*F*_v_/*F*_m_	ns	^***^
SUVAC	^***^	^**^
IUVAC	^***^	ns
SUVAC/IUVAC	^***^	^**^
*p*-Coumaric acid	^***^	^*^
Sclerophylly index	^***^	ns

Data are derived from a two-way ANOVA. Only the variables that were common to the three species are shown. SUVAC, total content of methanol-soluble UV-absorbing compounds; IUVAC, total content of methanol-insoluble UV-absorbing compounds. Three replicates for each variable were used. For each variable, significance levels for statistical analysis are shown:

*** p < 0.001;

** p < 0.01;

* *p < 0.05; ns, non-significant*.

**Figure 2:**
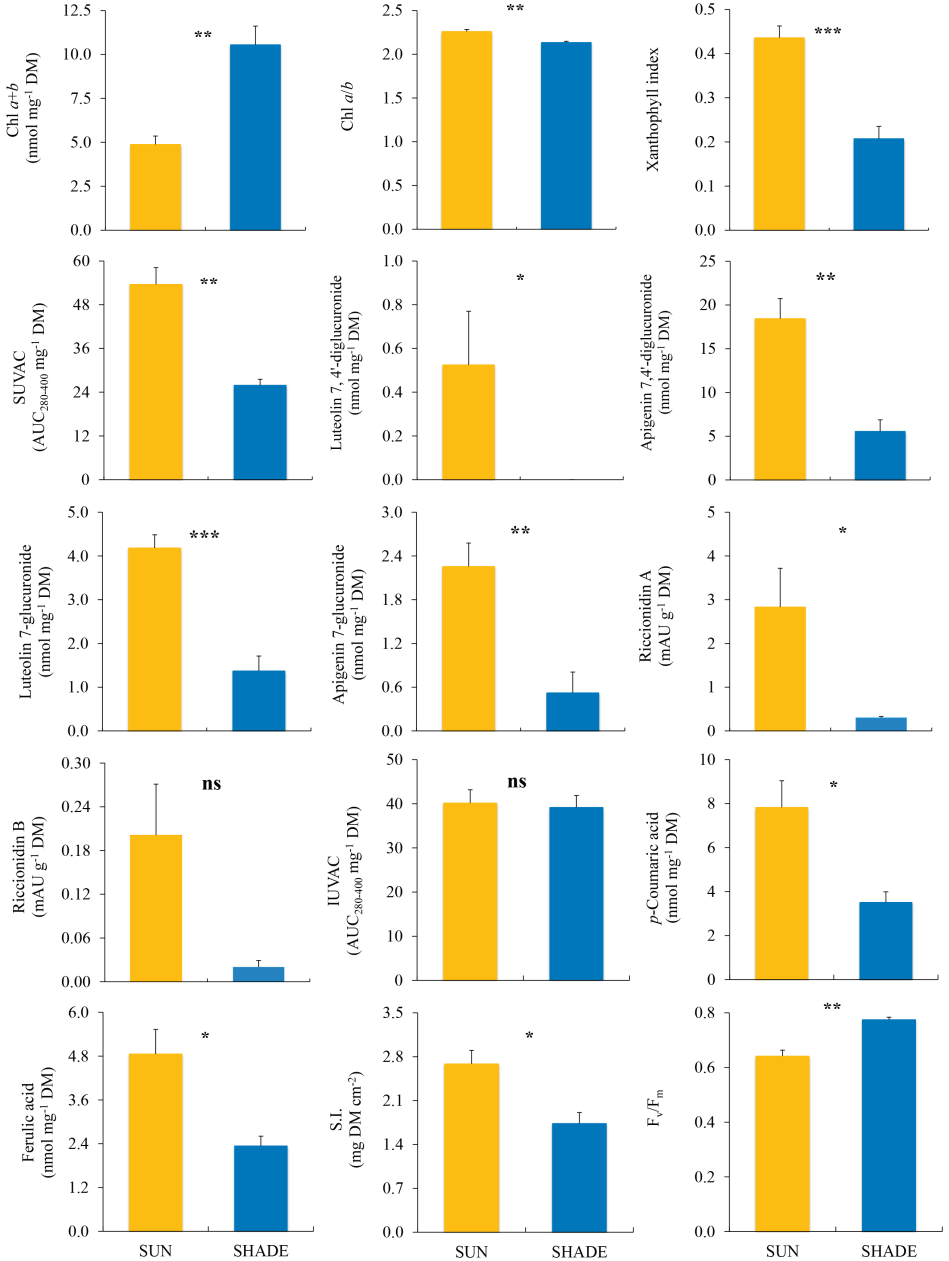
Comparison (mean ± SE, *n* = 3) of different variables measured in sun and shade samples of *Marchantia polymorpha* subsp. *polymorpha*. SUVAC and IUVAC, total contents of UV-absorbing compounds from the soluble and insoluble fractions, respectively, in terms of the area under the absorbance curve in the interval 280–400 nm (AUC_280–400_) per DM unit. SI, sclerophylly index. Significance levels for statistical analysis are shown: ^***^p < 0.001; ^**^p < 0.01; ^*^p < 0.05; ns, non-significant.

In *M. polymorpha*, all the variables related to the photosynthetic apparatus showed highly significant differences between the two exposures (Figure 2). Values of xanthophyll index and Chl *a*/*b* ratio were significantly higher in sun than in shade samples, while Chl *a* + *b* content and *F*_v_/*F*_m_ showed the opposite pattern. Regarding the variables related to secondary metabolism, the total content of SUVACs and the contents of every individual compound (except riccionidin B, which nevertheless showed a similar non-significant trend) were higher in sun than in shade samples. The contents of the individual IUVACs (C12 and C13) were significantly higher in sun than in shade samples, but there was no difference between them in the total contents of IUVACs. The SUVAC/IUVAC ratio was significantly higher in sun than in shade plants, but the luteolin/apigenin ratio did not show significant differences between both types of plants. SI was significantly higher in sun than in shade samples.

**Figure 3:**
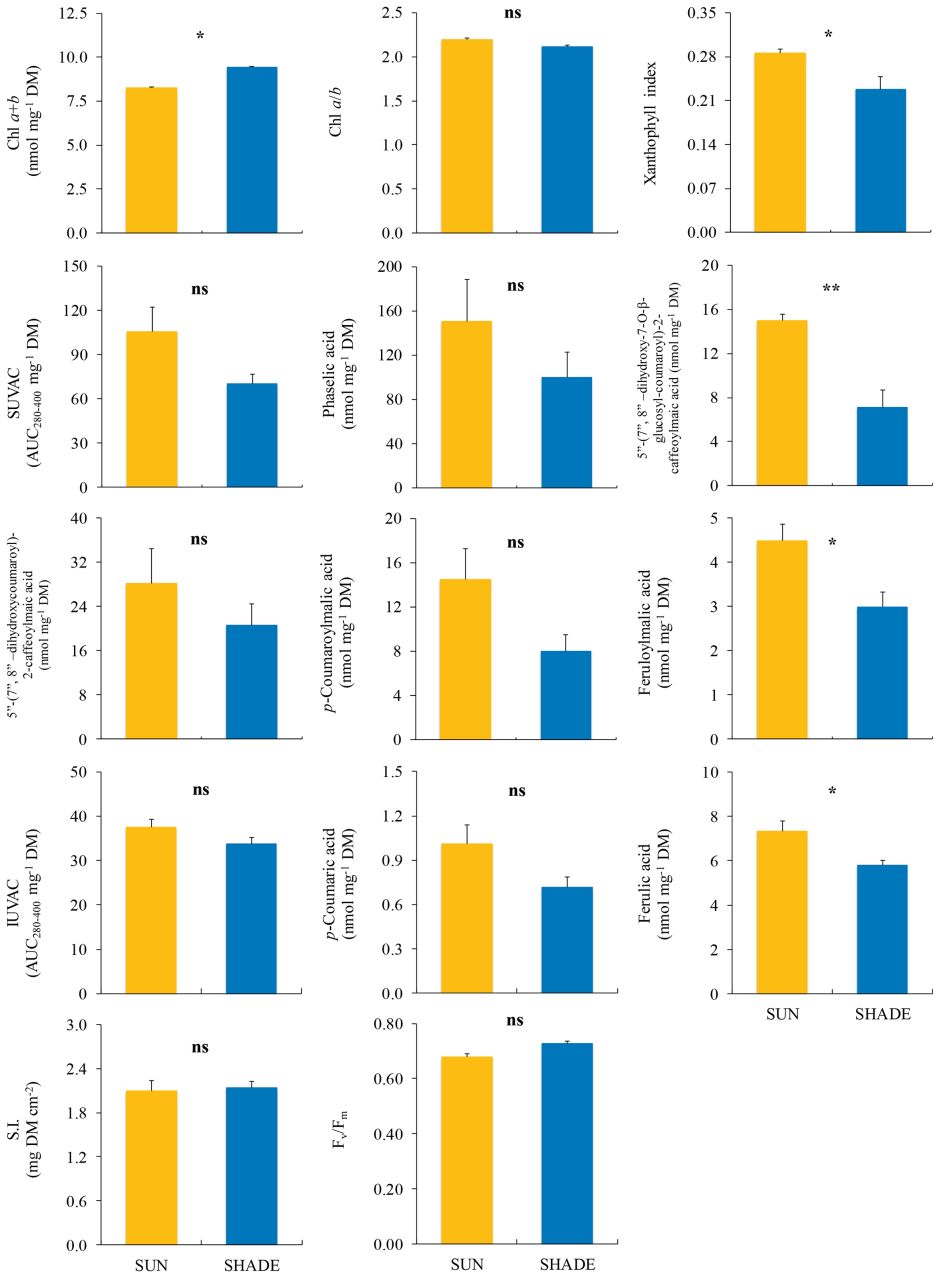
Comparison (mean ± SE, *n* = 3) of different variables measured in sun and shade samples of *Jungermannia exsertifolia* subsp. *cordifolia*. SUVAC and IUVAC, total contents of UV-absorbing compounds from the soluble and insoluble fractions, respectively, in terms of the area under the absorbance curve in the interval 280–400 nm (AUC_280–400_) per DM unit. SI, sclerophylly index. Significance levels for statistical analysis are shown: ^**^p < 0.01; ^*^p < 0.05; ns, non-significant.

In *J. exsertifolia* (Figure 3), only two variables related to the photosynthetic apparatus showed significant differences between sun and shade samples. Again, xanthophyll index was higher in sun than in shade samples, while Chl *a* + *b* content showed the opposite pattern. Regarding UVACs, only two individual SUVACs (C8 and C11) and one IUVAC (C13) showed significant differences between sun and shade samples, with higher values under the sun than in the shade. The remaining compounds, the total contents of SUVACs and IUVACs, and the SUVAC/IUVAC ratio, showed a similar non-significant trend. SI did not show any difference between sun and shade samples.

**Figure 4:**
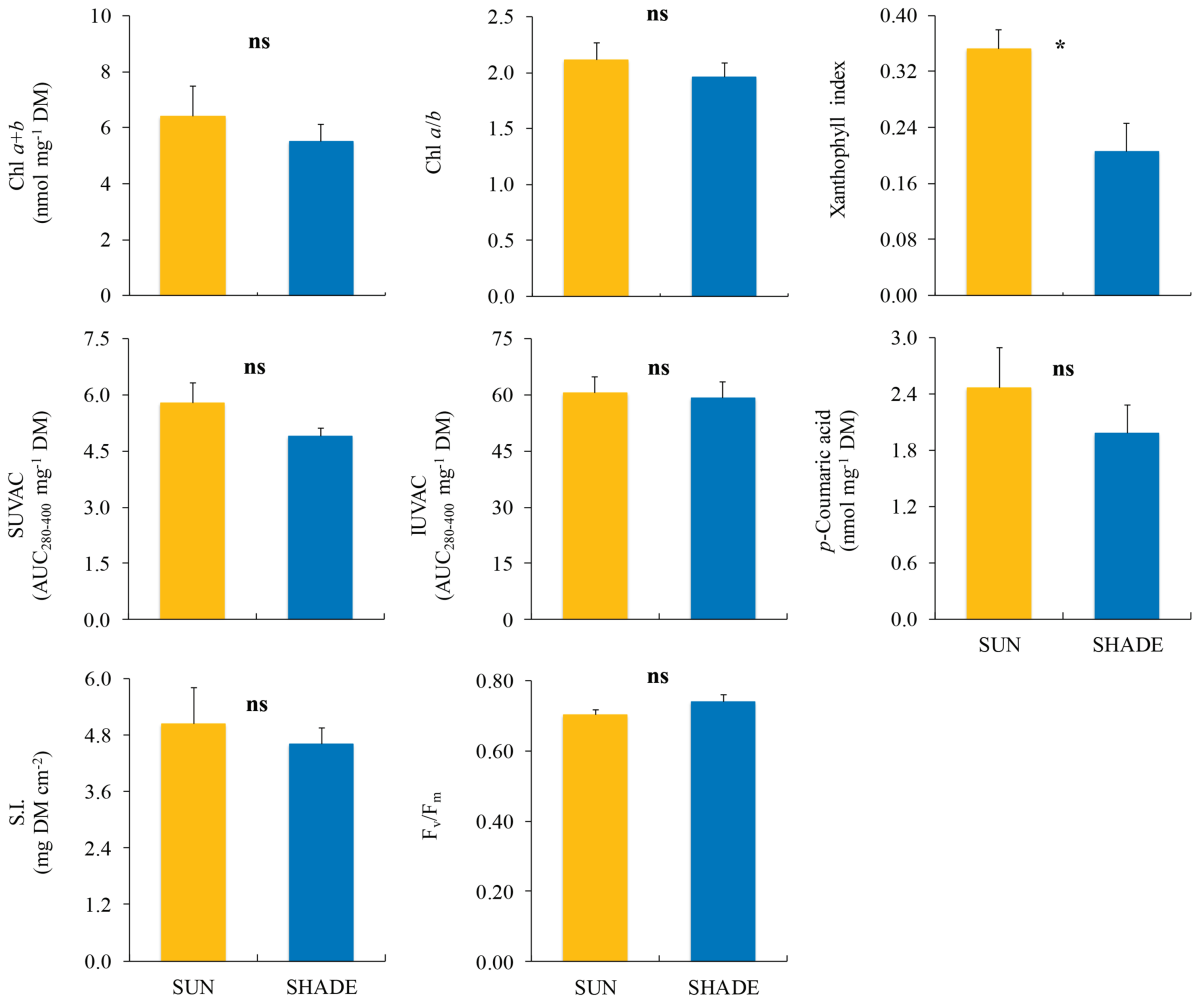
Comparison (mean ± SE, *n* = 3) of different variables measured in sun and shade samples of *Fontinalis antipyretica*. SUVAC and IUVAC, total contents of UV-absorbing compounds from the soluble and insoluble fractions, respectively, in terms of the area under the absorbance curve in the interval 280–400 nm (AUC_280–400_) per DM unit. SI, sclerophylly index. Significance levels for statistical analysis are shown: ^*^p < 0.05; ns, non-significant.

Among the variables measured in *F. antipyretica* (Figure 4), only the xanthophyll index showed significant differences between sun and shade samples, being higher under the sun than in the shade.

### Ordination of Field Samples by Principal Components Analysis

**Figure 5:**
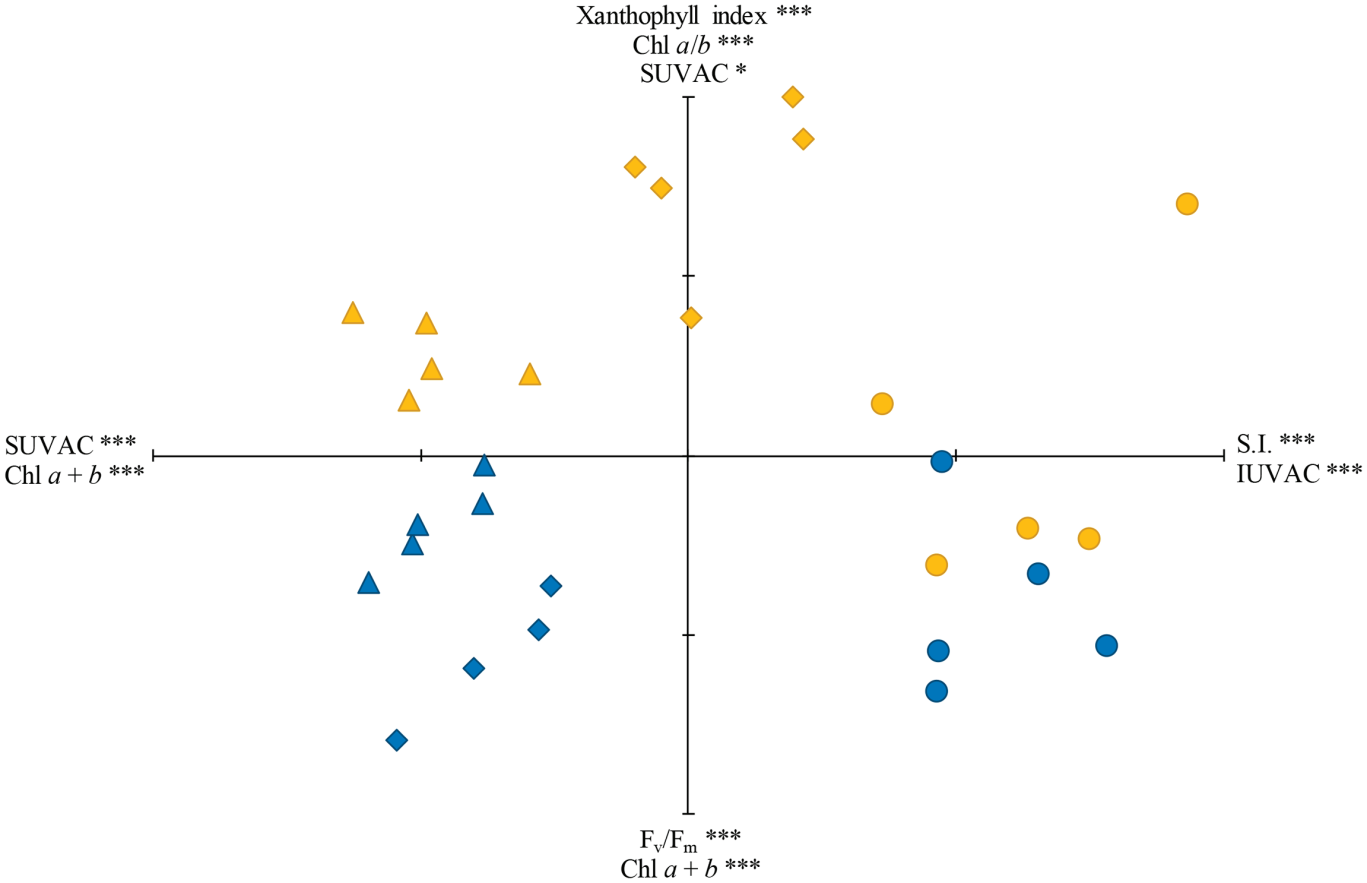
Ordination, through principal components analysis (PCA), of the sun (yellow) and shade (blue) samples of the three species studied: *Marchantia polymorpha* subsp. *polymorpha* (diamonds), *Jungermannia exsertifolia* subsp. *cordifolia* (triangles), and *Fontinalis antipyretica* (circles). SUVAC and IUVAC, total contents of UV-absorbing compounds from the soluble and insoluble fractions, respectively. SI, sclerophylly index. Significant loading factors for the positive and negative parts of each axis, together with their corresponding significance levels, are shown. ^***^p < 0.001; ^*^p < 0.05. Axis I is the horizontal one, and axis II is the vertical one. Each tick mark on axes I and II represents 1 U.

The samples studied were ordinated by PCA using the variables that were common to the three species studied. The accumulated variance in the first three axes was 81.4% (37.6% for axis I, 30.7% for axis II, and 13.1% for axis III). The plot using the first two axes, together with the loading factors and their respective significances, is shown in Figure 5. The total content of IUVACs and SI were significant loading factors for the positive part of axis I, whereas the loading factors for its negative part were the total content of SUVACs and Chl *a* + *b* content. This axis ordinated the samples attending mostly to the differences in the localization of UVACs in the methanol-soluble or -insoluble fraction, especially separating the moss from the liverworts. SI additionally contributed to separate the more sclerophyllous moss from the softer liverworts, although the sun samples of *M. polymorpha* were situated in an intermediate place. Regarding axis II, the significant loading factors for its positive part were xanthophyll index, Chl *a*/*b* ratio, and the total content of SUVACs, whereas *F*_v_/*F*_m_ and Chl *a* + *b* content were loading factors for the negative part. Sun and shade samples were mostly placed toward the positive and negative parts of axis II, respectively. This pattern was more defined for the liverworts than for the moss.

### Greenhouse Experiment Using *M. polymorpha* subsp. *polymorpha*

**Figure 6:**
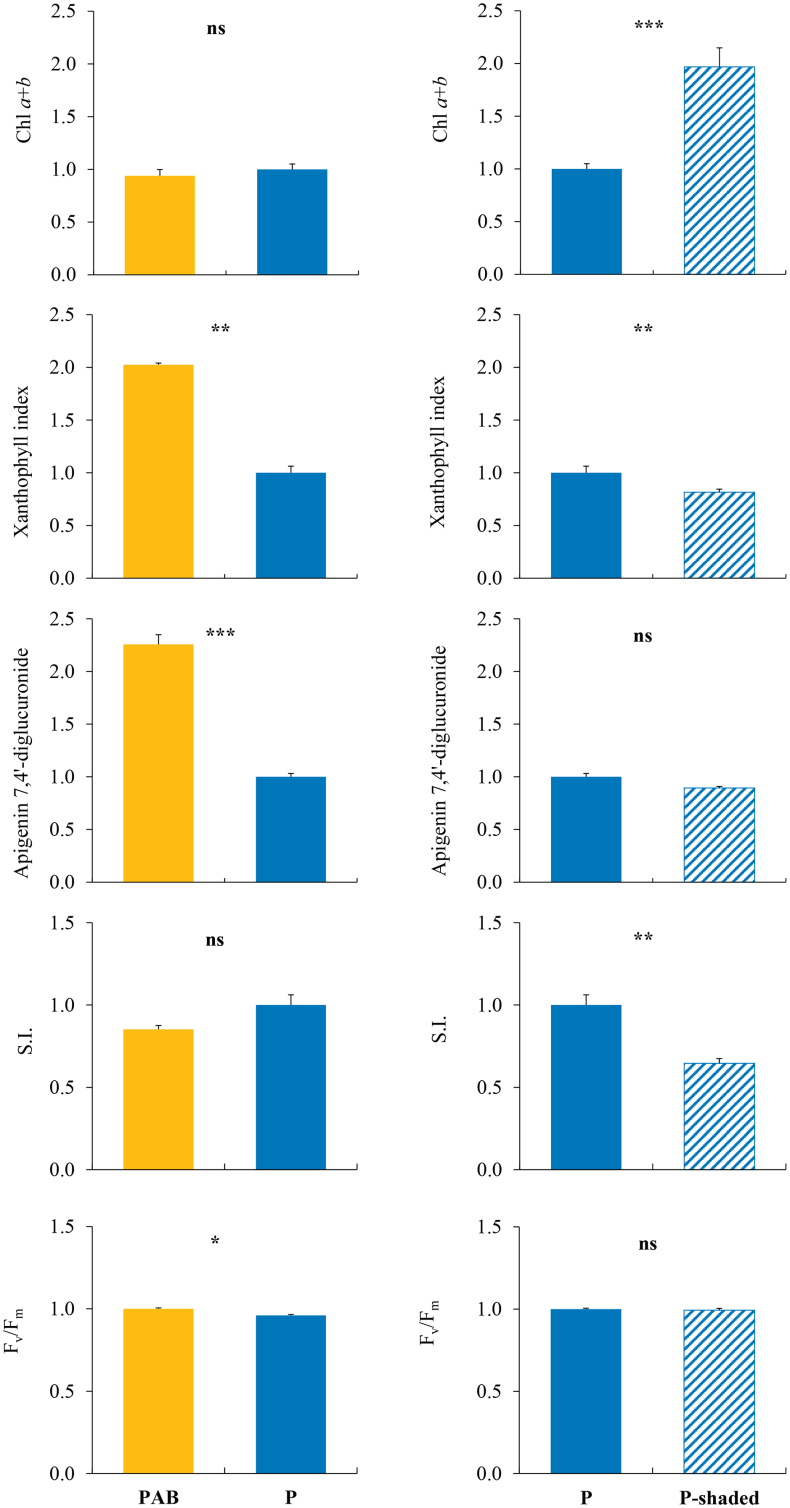
Variables which showed, in samples of *Marchantia polymorpha* subsp. *polymorpha* grown in the greenhouse experiment, significant differences between PAB and P treatments (effect of UV radiation, left column), or between P and P-shaded treatments (effect of PAR level, right column). P, PAR alone. P-shaded, reduced PAR using a shade cloth (see Figure 1, bottom). PAB, PAR + UV-A + UV-B. SI, sclerophylly index. All the results are expressed in relative units, taking the P treatment as the unit value. For each variable, means ± SE (*n* = 3), together with significance levels for statistical analysis, are shown: ^***^p < 0.001; ^**^p < 0.01; ^*^p < 0.05; ns, non-significant.

The significant separate effects of PAR level (P vs. P-shaded samples) and UV radiation (P vs. PAB samples) on the different variables measured are shown in Figure 6. Chl *a* + *b* content and SI were only affected by PAR level, with P samples showing lower Chl *a* + *b* contents and higher SI values than P-shaded samples. *F*_v_/*F*_m_ was only affected by UV radiation, with higher values in PAB than in P samples. Also, apigenin and luteolin derivatives were only affected by UV radiation (the most abundant derivative in *M. polymorpha*, C2, is shown as an example in Figure 6). The xanthophyll index was affected by both PAR (with higher values in P than in P-shaded samples) and UV radiation (with higher values in PAB than in P samples). The remaining variables did not show significant differences caused by neither PAR nor UV radiation.

## Discussion

### The Species Influences Pigment Contents and Responsiveness to Radiation Under Field Conditions

The species factor affected most of the variables assessed, in particular the contents of UVACs and Chl *a* + *b*. This was expected, because the species studied differed in several architectural and anatomical characteristics, such as the thalloid or leafy morphology, the proportion of leaves to stems in leafy species, and the sclerophylly (influenced by the proportion of supporting tissues, the proportion of cell walls to protoplasts, etc.). All these characteristics determine pigment contents through the influence on DM, surface area, and the proportion of photosynthetic and non-photosynthetic tissues (Martínez-Abaigar and Núñez-Olivera, 1998). For example, Chl *a* + *b* contents per DM in the moss were lower than in the liverworts, mainly because of the higher sclerophylly in the moss. This is in line with previous results obtained in bryophytes (Marschall and Proctor, 2004). In contrast to the pigment contents, two of the three ratios measured (the xanthophyll index and *F*_v_/*F*_m_) were not influenced by the species, probably because they are determined by the rather homogeneous physiological state of the photosynthetic apparatus and not by the gametophyte structure. The third ratio (Chl *a*/*b*) was slightly higher in the liverworts than in the moss but differences were small, as found by Marschall and Proctor (2004).

The species also influenced the responsiveness to the radiation environment. *M. polymorpha* was the most responsive species, showing the greatest differences between sun and shade samples, whereas *F. antipyretica* was the least responsive species and *J. exsertifolia* occupied an intermediate place. The causes underlying these interspecific differences may be diverse, and could be related to: (1) the fact of being a moss or a liverwort, because mosses are generally less responsive to radiation than liverworts (Rincón, 1993; Fabón et al., 2010, 2012a); (2) the bryophyte structure, with thalloid species showing a higher responsiveness than leafy species; and (3) the higher plasticity typical of early successional species such as *M. polymorpha*, as occurs in tracheophytes (Portes et al., 2010). More studies are needed to support these hypotheses.

### Responses of the Photosynthetic Apparatus to Sun and Shade in the Field

The most consistent response, which took place in the three species studied, was the increase in xanthophyll index in sun samples in comparison with shade samples. This response protected PSII from excess radiation through a nonphotochemical dissipation of energy due to the increase in deepoxidated forms of xanthophylls (Esteban et al., 2015). An excess of PAR and/or UV radiation can be responsible for this response, both in bryophytes (Robinson et al., 2005; Otero et al., 2006; Arróniz-Crespo et al., 2011; Schroeter et al., 2012; Fabón et al., 2012a; Soriano et al., 2019b) and tracheophytes (Krause et al., 2003; Guidi et al., 2011). Given that the xanthophyll index increases during the central hours of the day, thus increasing dissipation of excessively absorbed energy (Fabón et al., 2012b), the values measured would represent daily maximum values for the species and environmental conditions of the present study.

Other responses were species-specific. For example, Chl *a* + *b* content per DM was higher in shade than in sun samples only in the two liverworts, but not in the moss. This difference has been repeatedly found in bryophytes (Martin and Churchill, 1982; Glime, 1984; Post and Vesk, 1992; Rincón, 1993; Marschall and Proctor, 2004; Hooijmaijers and Gould, 2007; Hajek et al., 2009) and tracheophytes (Pearcy, 1998). The reasons underlying this difference may be multiple. A higher Chl content per DM in shade plants represents an adaptation to enhance the efficiency of light absorption in the shade (Martin and Churchill, 1982), but, at the same time, a lower content in sun plants can indicate an increased Chl degradation due to excessive PAR or UV radiation (Martínez-Abaigar and Núñez-Olivera, 1998; Robinson et al., 2005; Guidi et al., 2011; Soriano et al., 2019b). Another influencing factor could be sclerophylly. In both bryophytes and tracheophytes, the generally higher Chl *a* + *b* content per DM in shade plants has been related to a lower sclerophylly, which implies a decrease in DM per unit area (Martínez-Abaigar and Núñez-Olivera, 1998; Pearcy, 1998). This would be the reason why, in general, shade plants contain more Chl per unit mass than sun plants, but there exists no consistent response of Chl content per unit area basis to PAR level (Pearcy, 1998; Krause et al., 2003; Poorter et al., 2019). In our study, differences in Chl content between sun and shade plants were similar when considering both per DM and per surface area basis, and thus the influence of sclerophylly was not decisive. In fact, sun plants were more sclerophyllous than shade plants only in *M. polymorpha*. Thus, the higher Chl *a* + *b* content in shade than in sun samples that was found in the two liverworts would have been caused by an enhanced efficiency of light absorption in the shade combined with a stronger Chl degradation in the sun. The lack of response of Chl *a* + *b* content in the moss would be related to its general lower responsiveness to changing radiation.

Significant differences between sun and shade plants in Chl *a*/*b* ratio and *F*_v_/*F*_m_ were found only in *M. polymorpha*, although the remaining species showed a similar trend. Chl *a*/*b* ratio was higher in sun than in shade samples. This frequently occurs in bryophytes (Martin and Churchill, 1982; Rincón, 1993; Martínez-Abaigar et al., 1994; Marschall and Proctor, 2004; Hooijmaijers and Gould, 2007) and thacheophytes (Krause et al., 2003; Esteban et al., 2015; Poorter et al., 2019), but not always (Glime, 1984; Post and Vesk, 1992; Rincón, 1993; Hooijmaijers and Gould, 2007; Schroeter et al., 2012). The lack of response in these cases was probably due to random variation (Marschall and Proctor, 2004) or to the diverse interacting internal and environmental factors that can influence this variable apart from PAR level: tissue activity, senescence, stress, nitrogen saving, UV radiation, etc. (Evans, 1986; Martínez-Abaigar and Núñez-Olivera, 1998, 2011). Hence, elucidating the precise role of Chl *a*/*b* ratio in radiation acclimation in bryophytes needs further study.

*F*_v_/*F*_m_ showed lower values in sun than in shade plants. This could be expected because a decrease in *F*_v_/*F*_m_ indicates a greater photoinhibition under sun conditions (Maxwell and Johnson, 2000; Guidi et al., 2011; Poorter et al., 2019). A similar photoinhibitory process was found in *Sphagnum* species from open habitats (Hajek et al., 2009), but not in the Antarctic moss *Grimmia antarctici* (Robinson et al., 2005). Different responses can be explained by the specific sensitivity of the different species. *F*_v_/*F*_m_ can be affected by both PAR and UV radiation (Fabón et al., 2012a; Wargent et al., 2015), and particularly a decrease in *F*_v_/*F*_m_ is a common response of bryophytes to enhanced UV-B (Martínez-Abaigar and Núñez-Olivera, 2011). Thus, the decrease in sun plants of *M. polymorpha* (and the other species) could be due to both PAR and UV. Nevertheless, the *F*_v_/*F*_m_ values found in our study (0.68–0.77) were relatively high in comparison with those found in other studies on *J. exsertifolia* and *F. antipyretica* under field conditions, where they dropped to 0.60 (Núñez-Olivera et al., 2009, 2010). Therefore, given that *F*_v_/*F*_m_ can be interpreted as an indicator of physiological vitality (Maxwell and Johnson, 2000), the plants used in our study showed a good physiological state irrespective of their radiation exposure.

### Responses of Ultraviolet-Absorbing Compounds to Sun and Shade in the Field

In *M. polymorpha*, all the individual UVACs, both soluble and insoluble (except riccionidin B), together with the total contents of SUVACs, showed significantly higher values in sun than in shade plants. In *J. exsertifolia*, the responses of UVACs were less (or non-) significant, but the trend was similar. By contrast, UVACs of *F. antipyretica* were much less diverse and did not respond to radiation changes. Thus, the responses of UVACs to changing radiation depended on the species, as occurred with the variables related to the photosynthetic apparatus. This species-dependence was expected because it had previously been demonstrated that UVACs of two of the species studied (*J. exsertifolia* and *F. antipyretica*) responded differently to UV under both laboratory and field conditions, being more reactive in the liverwort than in the moss (Martínez-Abaigar et al., 2003; Núñez-Olivera et al., 2009, 2010; Fabón et al., 2010, 2012a). Regarding *M. polymorpha*, several studies have proved that some individual UVACs increase under enhanced UV, but using laboratory conditions and genotypes of a different subspecies of *M. polymorpha* (Albert et al., 2018; Soriano et al., 2019a,b). Nevertheless, Markham et al. (1998) did not find any significant change in UVAC contents when plants were exposed to enhanced UV-B, although the luteolin/apigenin ratio increased. In our study, the first performed under field conditions, we have demonstrated that the levels of most individual UVACs of *M. polymorpha* subsp. *polymorpha* increased under sun conditions.

The responses of the total contents of SUVACs and IUVACs to changing radiation were more diffuse than those of individual UVACs, probably because total contents integrate many different individual compounds with potentially diverse responses (positive, negative, or neutral) to radiation (Fabón et al., 2010, 2012a). In tracheophytes, total SUVACs can be stimulated by high levels of only PAR (Krause et al., 2003; Barnes et al., 2013; Laureau et al., 2015; Poorter et al., 2019). No comparable results have been obtained in bryophytes, but total SUVACs can increase (Newsham and Robinson, 2009; Núñez-Olivera et al., 2009, 2010) or remain unchanged (Robinson et al., 2005; Núñez-Olivera et al., 2010; Arróniz-Crespo et al., 2011) in response to increased UV. Thus, identification of individual UVACs is recommendable to find more defined responses to changing radiation.

The responses of SUVACs were generally clearer than those of IUVACs. Although the responses of IUVACs to changing radiation have been mostly ignored in the literature, the data available allow to relate this finding with the fact that IUVACs are relatively immobilized in the cell wall, which would limit their reactiveness (Fabón et al., 2010, 2012a). In addition, IUVACs seem to be rather constitutive while SUVACs would be more easily inducible (Monforte et al., 2018). The different responses of SUVACs and IUVACs were also influenced by the type of bryophyte considered, because the two liverworts, but not the moss, showed higher SUVAC/IUVAC ratios in sun than in shade samples. As it was commented above, liverworts are generally more radiation-responsive than mosses (Rincón, 1993; Fabón et al., 2010, 2012a). This can partially be due to the fact that the more radiation-responsive SUVACs are generally more abundant in liverworts than in mosses, whereas the less radiation-reactive IUVACs usually prevail in mosses (Monforte et al., 2018).

The individual soluble UVACs were species-specific, whereas the insoluble ones were mostly common to the three species studied. In *M. polymorpha*, the contents of the soluble luteolin and apigenin derivatives were higher in sun than in shade samples. No comparable field results exist for this species, but mostly coincident results were obtained in laboratory studies using several genotypes of *M. polymorpha* subsp. *ruderalis* exposed to enhanced UV radiation (Albert et al., 2018; Soriano et al., 2019a,b). Nevertheless, Markham et al. (1998) did not find any significant effect of high UV-B levels on luteolin and apigenin glycosides in an unidentified subspecies of *M. polymorpha*. In future research, it would be important to identify the material used at the subspecies and accession levels, because each genotype may have a different phenolic profile and may respond differently to radiation changes. Results obtained in tracheophytes are somewhat diverse, and can be summarized in that: (1) luteolins are generally induced by high sunlight, but the role of UV in this induction is uncertain and (2) the induction of apigenins by high sunlight is infrequent (Guidi et al., 2011; Agati et al., 2012; Brunetti et al., 2013). Taking into account all these findings, the higher contents of luteolins and apigenins that were found in our study in sun plants of *M. polymorpha* subsp. *polymorpha* could mainly be attributed to UV radiation. Regarding the luteolin/apigenin ratio, we found no difference between sun and shade plants, but it increased in *M. polymorpha* under high UV-B (Markham et al., 1998) and in tracheophytes in response to high UV-B, UV-B plus UV-A, or PAR irradiance (Brunetti et al., 2013). The differences outlined can be explained by genetic factors, probably related to the different regulation operating on flavonoid synthesis in each genetic entity (Albert et al., 2018), the acclimation history and developmental stage of each material, and the diverse experimental conditions used. The specific role that luteolins and apigenins play in *M. polymorpha* is still unknown. They could act as UV screens, according to their absorption maxima in the UV-A region, and as antioxidants, with luteolins showing a higher antioxidant capacity (due to their dihydroxy B-ring-substituted structure) than apigenins (which are monohydroxy B-ring-substituted forms).

The content of the soluble anthocyanidin riccionidin A increased in sun plants of *M. polymorpha*. Similar explanations have been found for this and other anthocyanidins in bryophytes, mainly liverworts, although it is not clear the solar wavelength responsible for induction (PAR, UV, or both), or if other factors, such as cold, are needed for this process (Glime, 1984; Hooijmaijers and Gould, 2007; Snell et al., 2009). Anthocyanidins may play a role in photoprotection (Robinson and Waterman, 2014).

The individual insoluble UVACs of *M. polymorpha* (*p*-coumaric and ferulic acids) responded similarly to the soluble UVACs, being higher in sun than in shade plants. In experiments under controlled conditions, neither *p*-coumaric nor ferulic acids were significantly induced by UV in two different accessions of subsp. *ruderalis* and, in one of them, their contents were higher under PAR than under UV (Soriano et al., 2019a,b). In some tracheophytes, hydroxycinnamic acids were higher in sun than in shade leaves (Kolb et al., 2001; Jaakola et al., 2004), which was generally attributed to PAR and not UV wavelengths, but other studies point out that these compounds were unresponsive to light (Agati et al., 2011). Again, genetic and experimental differences may explain these discrepancies. Future studies should take into account that: (1) hydroxycinnamic acids may preferentially be located bound to the cell walls, which makes recommendable to analyze both the soluble and insoluble UVACs and (2) some hydroxycinnamic acids are flavonoid precursors, and thus their increase in sun plants could just reflect the overall activation of flavonoid biosynthesis (Jaakola et al., 2004).

In *J. exsertifolia*, only three UVACs showed significantly higher values in sun than in shade plants, but the remaining four compounds showed a similar, although non-significant, trend. This is congruent with the high responsiveness of the UVACs of this species to UV radiation under both controlled and field conditions (Núñez-Olivera et al., 2009; Martínez-Abaigar and Núñez-Olivera, 2011; Monforte et al., 2015).

In *F. antipyretica*, the only UVAC found (*p*-coumaric acid in the insoluble fraction) did not show any significant difference between sun and shade plants. This was not surprising because, although the studies available only considered the influence of UV and not PAR, *F. antipyretica* usually did not respond to changing radiation, either under field or laboratory conditions (Núñez-Olivera et al., 2010; Fabón et al., 2012a). In general, mosses and IUVACs are less radiation-responsive than liverworts and SUVACs, respectively.

### Responses of Sclerophylly Index to Sun and Shade in the Field

SI responded only in *M. polymorpha*, with higher values in sun than in shade plants. This was already found in bryophytes under field conditions, without further discussion on the solar wavelengths responsible for this morphostructural change (Glime, 1984; Martínez-Abaigar et al., 1994; Monforte et al., 2018). In other cases, the SI increase was specifically caused by enhanced UV-B radiation (Arróniz-Crespo et al., 2011; Martínez-Abaigar and Núñez-Olivera, 2011). In *M. polymorpha* subsp. *ruderalis* cultivated under controlled conditions, SI only increased when plants were exposed to the sum of PAR, UV-B, and UV-A radiation, in comparison with plants exposed to only PAR or PAR plus UV-A (Soriano et al., 2019b). Thus, UV-B seems to play a role in the SI increase, which could represent a protecting mechanism against UV through increasing the path which radiation must cross to reach potential targets within the cells. Also, the higher sclerophylly reported under enhanced UV in bryophytes may be due to a lower elongation which could lead to the production of harder tissues. In tracheophytes, SI increased under high sun exposures (Pearcy, 1998; Poorter et al., 2019), in line with the results found in *M. polymorpha*. The lack of SI response in *J. exsertifolia* and *F. antipyretica* could be due to a lower structural plasticity of their leafy shoots in comparison with *M. polymorpha* thalli. However, at least in some experiments, SI increased in *J. exsertifolia* and *F. antipyretica* under enhanced UV-B or under sun conditions (Glime, 1984; Martínez-Abaigar et al., 2003). These findings demonstrate a certain structural plasticity in the three bryophytes studied, but the specific wavelength causing their SI changes is not perfectly known.

### Principal Components Analysis Summarized the Acclimation Modalities to Sun or Shade in the Field

The PCA plot (Figure 5) reliably summarized the acclimation modalities of the three species to sun or shade conditions, which were determined by PAR and UV radiation. Axis I was mainly defined by variables which, according to the literature available, mostly respond to UV radiation (in particular, SUVACs and IUVACs contents), whereas variables defining axis II were predominantly related to the photosynthetic apparatus and, presumably, were mostly affected by PAR but also by UV (mainly xanthophyll index, *F*_v_/*F*_m_, Chl *a* + *b* content, and Chl *a*/*b* ratio). Overlapped over this frame of variables, there exists an additional variable (SI) which may respond to both PAR and UV radiation. In this scenario, the moss *F. antipyretica* was sharply separated from the two liverworts along axis I, because of its higher proportion of IUVACs than SUVACs and higher sclerophylly, both of which are typical characteristics of mosses (Monforte et al., 2018). In relation to axis II, the moss showed the least defined model of radiation acclimation among the three species studied, because the sun and shade plants rather appeared in a diffuse mixed group than in two compact separate groups as occurred in the liverworts. This was in line with the fact that *F. antipyretica* was the least responsive species to radiation changes. The liverwort *J. exsertifolia* showed an intermediate radiation acclimation capacity in our study. Regarding UV responses (axis I), it adopted the model typical of liverworts, with a higher proportion of SUVACs than IUVACs, but UVACs contents were not very different between sun and shade plants, thus showing a limited acclimation capacity to UV. This was surprising because *J. exsertifolia* was generally a UV-responsive species under both laboratory and field conditions (Núñez-Olivera et al., 2009; Fabón et al., 2010; Monforte et al., 2015). Regarding the PAR responses of this liverwort (axis II), sun and shade plants were grouped in two clearly separated but close groups, thus not being very sharply contrasted. In fact, only two variables related to the photosynthetic apparatus (xanthophyll index and Chl *a* + *b* content) were significantly different between sun and shade plants. *M. polymorpha* was the most radiation-responsive species, with sun and shade plants most distant along both axes. Regarding axis I, *M. polymorpha* sun plants were clearly separated from both *J. cordifolia* plants and *M. polymorpha* shade plants by a combination of characteristics rather peculiar for a liverwort: unusually low values of the SUVAC/IUVAC ratio and high values of SI, and low Chl *a* + *b* content. Regarding axis II, *M. polymorpha* sun plants showed the typical responses to high PAR levels: low *F*_v_/*F*_m_ (denoting photoinhibition), low Chl *a* + *b* content to reduce light absorption and alleviate overexcitation, and high values of the xanthophyll index to provide photoprotection through energy dissipation.

### Influence of Photosynthetically Active and Ultraviolet Radiations on *Marchantia polymorpha* subsp. *polymorpha* Acclimation

In the field, *M. polymorpha* subsp. *polymorpha* showed diverse responses to sun and shade conditions that could be attributed to PAR and/or UV radiation. These responses were associated with variables related to both the photosynthetic apparatus and UV protection. Given that this liverwort was the most radiation-responsive in the field, the separate influence of PAR and UV on those variables was tested under close-to-ambient conditions (greenhouse), where some variables responded only to PAR, others only to UV, and others to both or none of those radiation types. Considering all this variability, we tried to match field and greenhouse results for the variables measured.

In the field, xanthophyll index increased in sun samples in comparison with shade samples, which was attributed (as derived from the literature) to an excess of PAR and/or UV radiation. In the greenhouse, it was confirmed that both PAR and UV were responsible for this increase. Results were also conclusive for the contents of apigenin and luteolin derivatives. In the field, these contents were higher in sun samples, which was attributed to enhanced UV. In the greenhouse, they were specifically induced by UV radiation, but not by PAR. Thus, these compounds would increase to provide protection against UV excess, on the basis of their role as antioxidants and UV screens. Another variable showing congruent results between field observations and the greenhouse experiment was total IUVACs, because it did not react to radiation changes in any case.

The remaining variables showed inconclusive results. The increase of total SUVACs and riccionidin A in sun samples in the field could be attributed mainly to UV, or to both PAR and UV, respectively. However, these two variables were not affected by any type of radiation in the greenhouse. Regarding individual IUVACs (*p*-coumaric and ferulic acids), literature data supported that their increase in sun samples in the field could rather be due to PAR than UV. However, neither PAR nor UV affected these compounds in the greenhouse. On the basis of the literature available, the Chl *a* + *b* content decrease in sun samples in the field could be due to both PAR and UV, but in the greenhouse it was only affected by PAR, always showing higher values in the shade. Thus, the influence of UV radiation on Chl degradation remains elusive. The Chl *a*/*b* ratio responded to radiation in the field, probably showing the influence of PAR, UV, and other factors, but it did not respond in the greenhouse. According to a vast literature, the *F*_v_/*F*_m_ decrease we detected in sun samples in the field, indicating photoinhibition, could have been caused by high PAR or high UV radiation, but *F*_v_/*F*_m_ was only affected by UV radiation in the greenhouse. In addition, it was surprising that samples exposed to PAR + UV were less photoinhibited than samples exposed to only PAR. Finally, SI increased in sun samples in the field, which could be attributed to both UV and PAR, but SI was only influenced by PAR in the greenhouse. All these inconclusive results can be explained by the diverse factors (not only PAR and UV radiation) influencing these variables, and by the different experimental conditions used in the different studies.

In conclusion, different bryophytes show specific response strategies to sun and shade conditions, and both PAR and UV radiation are important to better interpret the bryophyte responses to changing radiation in the field. In addition, more research is needed, especially under ambient conditions, to explain the inconclusive results found for some variables regarding the role that PAR and UV may play in the radiation acclimation of bryophytes.

## Data Availability

The datasets generated for this study are available on request to the corresponding author.

## Author Contributions

EN-O and JM-A conceived the study, designed the experimental setup, and wrote the manuscript with contributions from all the authors. All the authors selected the field populations, collected the samples, measured the variables included in the study, and discussed the results. JM-A and EN-O wrote the manuscript with contributions from all the authors

### Conflict of Interest Statement

The authors declare that the research was conducted in the absence of any commercial or financial relationships that could be construed as a potential conflict of interest.
